# Characterizing asset-based studies in public health: development of a framework

**DOI:** 10.1093/heapro/daad015

**Published:** 2023-03-18

**Authors:** Jacqueline Martin-Kerry, Jennifer McLean, Trevor Hopkins, Antony Morgan, Laurie Dunn, Robert Walton, Su Golder, Tim Allison, Des Cooper, Pia Wohland, Stephanie L Prady

**Affiliations:** School of Healthcare, University of Leicester, Leicester, UK; Glasgow Centre for Population Health, Glasgow, UK; Asset Based Consulting, Tyne and Wear, UK; Yunus Centre for Social Business and Health, Glasgow Caledonian University, London, UK; Hull York Medical School, University of Hull, Hull, UK; Centre for Primary Care and Public Health, Queen Mary University, London, UK; Department of Health Sciences, University of York, York, UK; Director of Public Health and Policy, NHS Highland, UK; Public Health Intelligence Hull City Council, Hull, UK; School of Earth and Environmental Sciences, University of Queensland, Brisbane, Australia; Department of Health Sciences, University of York, York, UK

**Keywords:** asset-based approaches, deficit-based approaches, characterization, framework, public health

## Abstract

Asset-based approaches are becoming more common within public health interventions; however, due to variations in terminology, it can be difficult to identify asset-based approaches. The study aimed to develop and test a framework that could distinguish between asset-based and deficit-based community studies, whilst acknowledging there is a continuum of approaches. Literature about asset-based and deficit-based approaches were reviewed and a framework was developed based on the Theory of Change model. A scoring system was developed for each of the five elements in the framework based on this model. Measurement of community engagement was built in, and a way of capturing how much the study involved an asset approach. The framework was tested on 13 studies examining community-based interventions to investigate whether it could characterize asset-based versus deficit-based studies. The framework demonstrated how much the principles underpinning asset-based approaches were present and distinguished between studies where the approach was deficit-based to those that had some elements of an asset-based approach. This framework is useful for researchers and policymakers when determining how much of an intervention is asset-based and identifying which elements of asset-based approaches lead to an intervention working.

## INTRODUCTION

Recently, there has been a shift in the way public health interventions are developed and implemented, moving from deficit-based approaches towards asset-based approaches. A deficit-based approach identifies problems or needs (deficiencies) and ‘solutions’ developed through resources that are often external to the community (Morgan and Ziglio, 2007). Whereas an asset-based approach aims to identify and use existing community assets, or strengths, to enable its members to have more control over their health and wellbeing (McLean, 2012; Rippon and Hopkins, 2015). This approach aims to appreciate and value connections and potential within a community rather than simply identifying and responding to problems and needs (Foot and Hopkins, 2010). In this way, the asset-based approach has been eloquently described as seeing the world as a ‘glass half full’ (Foot and Hopkins, 2010). In a health context, an asset is any resource that improves an individual’s or community’s ability to maintain good health, wellbeing and reduce inequities; for example, people’s skills, knowledge or resources (Morgan and Ziglio, 2007). These are framed within the context of the determinants of health paradigm rather than the determinants of disease. Asset-based approaches focus on the concept of salutogenesis as a means of guiding practice, where individuals’ successes, rather than failures, are emphasised. However, an ‘asset-based approach’ is not prescriptive or fixed, and can be informal (Evaluation Support Scotland, 2017), flexible reflecting a spectrum of activity (McLean, 2015) which takes into account the context of an individual’s life and their community as part of the process of health promotion (McLean and McNeice, 2012). A review of asset-based approaches applied to service mapping found that there were no specific or standardized guidelines on what the process entails (Green *et al.*, 2017). A recent report noted that working in an asset-based way may take many forms and cannot be set down, scaled up or rolled out. Asset-based approaches are context-specific and about people and relationships in a place and time (McLean *et al.*, 2017).

**Fig. 1:**
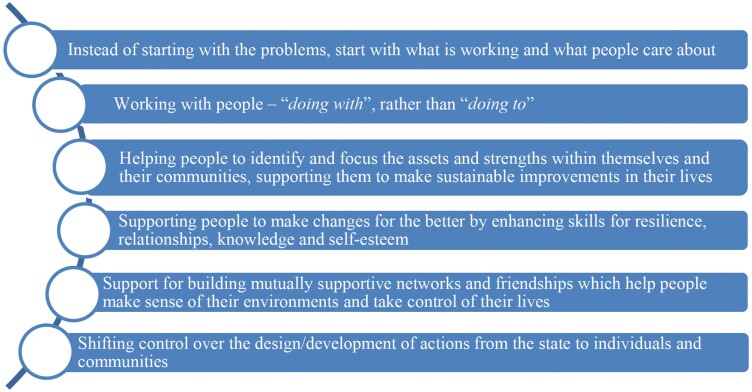
Principles of asset-based approaches (Reproduced from: Positive conversations, meaningful change: learning from Animating Assets, GCPH and SCDC, McLean, 2015; adapted from Foot and Hopkins, 2010).

The ‘asset-based approach’ has been described in numerous ways, utilizing varying terminology to express key elements involved (Morgan and Ziglio, 2007; Foot and Hopkins, 2010; Nurture Development, 2018). The Asset-Based Community Development (ABCD) approach, developed in the United States in the early 1990s, describes five stages, with a key feature being a focus on a community’s and its individuals’ strengths or assets (Kretzmann and McKnight, 1996; Nurture Development, 2018). Morgan and Ziglio in 2007 described an asset model as a way of facilitating a shift in emphasis for public health drawing on the concept of salutogenesis to focus attention on ‘health creation’ as compared to the ‘pathogenic focus on disease generation’ within deficit approaches (Morgan and Ziglio, 2007). It emphasized the importance of asset mapping, described within the ABCD model, as a way of positively engaging the community to identify strengths that could enhance their health. Importantly, asset-based approaches are underpinned by a set of values and principles, and a way of thinking (see Figure 1).

**Fig. 2:**
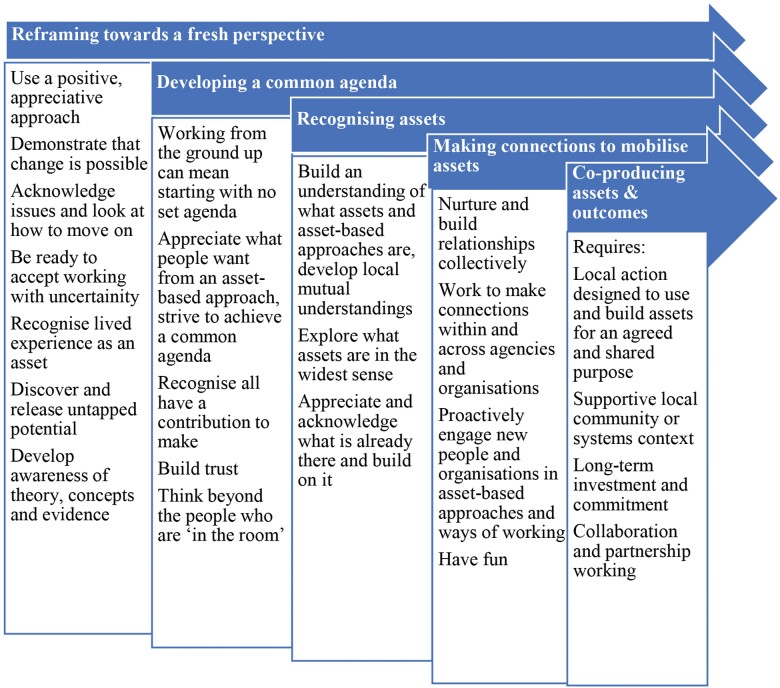
Theory of change for asset-based working (Reproduced from: Positive conversations, meaningful change: learning from Animating Assets, GCPH and SCDC, McLean, 2015).

Recent approaches to describing asset-based models in the UK have been based on the need for a ‘Theory of Change’ (Foot and Hopkins, 2010; McLean and McNeice, 2012; Rippon and Hopkins, 2015). Rippon and Hopkins proposed four elements needed for asset-based change to occur: ‘reframing towards assets’, ‘recognizing assets’, ‘mobilizing assets’ and ‘co-producing assets and outcomes’, encompassing the ‘head, hands and heart approach: what do people know, what can they do and what do they care about?’ (Rippon and Hopkins, 2015). This model was later adapted to suit community change initiatives and included an additional element: ‘developing a common agenda’ (McLean, 2015; see Figure 2). This modification illustrated that the five elements need not occur sequentially but can overlap and occur in parallel (McLean, 2015).

There are also differences of opinion in how asset-based approaches can interact with a deficit orientation (Kretzmann and McKnight, 1996). The ABCD approach cautions against using a deficit model. However, others recognize that deficit (perhaps more directive) approaches are necessary in some situations when problems need to be understood and resolved promptly (Morgan, 2014). Asset-based approaches take longer but can secure sustainable solutions to issues prioritized by communities. As such asset-based approaches can be seen as complementary to, not in competition with, deficit approaches (Morgan and Ziglio, 2007; Evaluation Support Scotland, 2017). There is a disproportionate amount of public health evidence available on how best to improve health that focuses on deficits as a starting point (Morgan and Ziglio, 2007), possibly because many asset-based approaches are not evaluated as they do not generally embrace research designs such as randomized controlled trials (Green *et al.*, 2017). Evidence of the effectiveness of asset-based approaches is more likely to be reported through case studies which may not capture similar outcomes to enable evaluation to be undertaken (Friedli, 2013; Green *et al.*, 2017).

Despite variations in how the approach is described, the common thread in an asset approach ‘values the capacity, skills, knowledge, connections and potential in a community’ (McLean, 2011), recognizing and valuing contributions that people have to offer (Foot and Hopkins, 2010). However, some studies (Leven, 2018) described as asset-based or community-based in approach may actually be more aligned to a deficit approach or may include only some elements of an asset-based approach such as recognizing or identifying assets, without the community mobilizing and co-producing these. A recent review assessing asset-based approaches to service mapping noted that, despite it being considered central to involve the community in traditional community asset mapping, the community identified the issue to be addressed in fewer than half of the studies described as asset-based (Green *et al.*, 2017). This highlights a debated idea between asset-based practitioners. Some believe needs identification sits firmly within a deficit-based approach, but needs can emerge when using an asset-based approach and should be addressed, alongside the recognition, mobilization and co-production of assets. Additionally, some studies using asset-based approaches may not use the term ‘assets’ or ‘asset-based approach’ (Fredland, 2010; Cassetti *et al.*, 2020), describing their process as ‘community engagement, community development or community-led’ (McLean, 2012). ‘Strengths-based approach’ is also used particularly within the social services field (Pattoni, 2012). In part, it is the inconsistency in the language used to describe the ideas behind the notion of ‘assets’ that has slowed its pace of development in mainstream public health.

A review exploring key characteristics of asset-based approaches to improve health within communities identified three characteristics: ‘connecting assets’, ‘raising awareness of available assets’ and ‘enabling assets to thrive’ (Cassetti *et al.*, 2020). Their focus was identifying characteristics of asset-based interventions that could be used within a framework to evaluate how asset-based approaches work and how assets are mobilized.

Synthesis of community-based projects for evidence of effectiveness is challenging and reviewers have innovated in operationalizing complex features, such as the degree of community engagement. However, it remains unclear which components of a community-based project led to the effectiveness of an intervention. Synthesis of asset-based studies is further challenged by the diversity of organizing models, their foci and the labelling of activities that might not accurately reflect the approach taken.

The aim of this article is to describe the development and implementation of a framework that distinguishes between asset-based and deficit-based studies whilst being sensitive to the diversity of asset-based models and recognizing that there is a continuum of approaches from deficit to asset-based, with each being useful depending on context. The framework can make an important contribution to distinguishing studies which are more aligned with an asset or a deficit-based approach. It will be useful for those who wish to distinguish between the overall study approach and the components within it.

## METHODS

An iterative approach was taken to the framework development: defining, classifying, testing and refining. A literature search was undertaken to see how asset-based approaches were described using the terms ‘asset-based approaches’, ‘assets’, ‘deficit approach’, ‘community engagement’, ‘community development’ and ‘community-led’. Searches were undertaken in PubMed and also in web searches due to the fact that many asset-based studies are not published in peer-reviewed journals. These can be developed, adopted and embedded in a range of ways but there are essential elements and framing within these approaches (McLean and McNeice, 2012; McLean *et al.*, 2017). The intention was that the framework would acknowledge this variation.

### Developing the framework

Key elements that the framework sought to capture were identified. The framework was based on the ‘Theory of Change’ model for asset-based approaches in community change initiatives (McLean, 2015). This model demonstrates the stages involved in asset-based change across a range of settings, taking a staged, iterative and action-focused approach. Each stage of the Theory of Change model was incorporated as a separate element within the framework. Variation in how each element is undertaken was allowed, with descriptions based on those within the asset-based approach literature. As acknowledged earlier, some projects described as an asset-based approach are not. Some that embrace an asset-based approach may not use terms such as ‘mobilization’, ‘assets’ or ‘co-production’. It was important to distinguish between asset-based and deficit-based studies and to classify studies that incorporated elements of both approaches.

### Scoring

The framework was designed to enable scoring of a range of levels within each element (e.g. fully/mostly/somewhat/absent) rather than a binary (present/absent) scale. Scoring is designed to be compatible with fuzzy-set qualitative comparative analysis (QCA) (Ragin, 1987; Longest and Vaisey, 2008; Rihoux and Lobe, 2009) but could be adapted. Fuzzy set QCA is a methodology looking at components within interventions to identify which are necessary or sufficient for an outcome to occur (Rihoux and Lobe, 2009; Rihoux *et al.*, 2011). It was expected that some interventions may have multiple components, with only a proportion of activities related to an asset-based approach. For example, a sexual health clinic could be set up at the same time as an asset-based intervention, independent of any community planning. As this may be an important factor when evaluating overall effectiveness, this was included as a weighing scale. Scoring this element could be omitted if programme evaluation is not the goal.

### Testing the framework

After developing the framework and scoring system, the framework was tested using community-based studies identified through a review about community engagement for health (Brunton *et al.*, 2014), a review of health assets in a global context (Van Bortel *et al.*, 2019) and from our comparative review of community-based studies looking at addressing smoking and tobacco use (Martin-Kerry *et al.*, 2019). One author (J.M.K.; a researcher) used the framework to test 13 papers of community-based studies to see whether it could distinguish between asset-based and deficit-based approaches. By having one person score each paper, this supported consistency in scoring and enabled direct comparison of scores between studies. The scoring results were discussed and approved by co-authors.

## RESULTS

### The development of the framework

Based on the Theory of Change model, the five elements of the model were developed. Table 1 demonstrates the framework and associated scoring.

**Table 1:** Framework for distinguishing asset-based studies from other community-based studies

Element	Description of ideal asset-based approach	Classification	Further step studies which score >0 for a concept
			Community involvement
*1.Reframing towards a fresh perspective (or towards assets)*	Changing the focus to be about what people care about within the community instead of focusing on needs or problems.	Whether reframing towards an asset approach has happened and is described: 1 = The focus is on what people care about 0.66 = Most of the focus is on what people care about 0.22 = Some of the focus is on what people care about with most of the focus being on needs or problems 0 = The focus is solely on needs or problems	1 = Community leads/owns this reframing process. 0.66 = Community collaborates in this reframing process 0.44 = Community is consulted during this reframing process 0.22 = Community is informed about this reframing process 0 = Community is not involved in the reframing process or not applicable (no reframing occurred)
*2. Developing a common agenda*	There may be no set agenda to begin with but over time a realistic and achievable shared agenda will develop. A discussion about what is possible and what people care about will occur. It is recognized that everyone has a contribution to make. Trust will be built and a broader thinking about who could help the community to achieve their goals.	Whether a shared agenda is mentioned, trust is built and there is a broader thinking about who could help in achieving the desired goals. 1 = A shared agenda is described, trust is built and there is a broad understanding of who could help 0.66 = Some discussion about a shared agenda and/or trust being built and/or recognition of who could help 0.22 = Limited description of a shared agenda, or trust being built or a description of thinking about who could help 0 = There is no description of a shared agenda, or trust being built or no description of thinking about who could help	1 = Community leads/owns this common agenda development 0.66 = Community collaborates in this common agenda development 0.44 = Community is consulted during this process 0.22 = Community is informed about this process 0 = Community is not involved or not applicable (no development of a common agenda occurred)
*3. Recognizing assets*	Were individual, community or institutional assets recognized? Description about assets which could include people’s enthusiasm or energy to get involved, skills, expertise, knowledge, organizations, etc. Look for identifying previously hidden assets. Look for an acknowledgment of what already exists and this being built on. Mapping may be mentioned in terms of an inventory of assets and relationships between these.	Descriptions of the ‘assets’ 1 = Yes, assets were extensively described and built on, including identifying previously hidden assets 0.66 = Yes, asset recognition was less extensively described 0.22 = Minimal attempt at describing the recognition of assets or it seems not fully undertaken 0 = No assets recognized/reported	1 = Community leads/owns this asset recognition process. 0.66 = Community collaborates in this process 0.44 = Community is consulted during this process 0.22 = Community is informed about this process 0 = Community is not involved in the recognition of assets or not applicable (no recognition of assets occurred)
*4. Making connections to mobilize assets*	Were the identified assets then supported and nurtured to grow? How existing relationships and connections were nurtured and how new ones were encouraged. Look for celebration of successes and building on this.	The description of existing and new connections and nurturing growth 1 = Yes, assets were clearly supported through existing and new connections and nurtured to grow 0.66 = Some support of assets was made with some connections made and some growth was evident 0.22 = Support for growing and nurturing assets was limited and/or not completed 0 = Support for growing and nurturing assets was absent or not described	1 = Community leads how the assets are nurtured and grown 0.66 = Community collaborates in how the assets are nurtured and grown 0.44 = Community is consulted about how the assets are nurtured and grown 0.22 = Community is informed about how the assets are nurtured and grown 0 = Community is not involved about how the assets are nurtured and grown or not applicable (there was no connections made to mobilize assets)
*5. Mobilizing and co-producing assets and outcomes*	Were assets mobilized for the agreed purpose? Whether the assets were used for their intended purpose.	1 = Yes, mobilization was clear and complete; assets were used for their intended purpose 0.66 = Mobilization was undertaken to some extent and most assets were used for their intended purpose was achieved 0.22 = Mobilization was limited and not complete or incompletely described; assets were used for their intended purpose in a very limited way 0 = Absent or not described **If this is zero, then regardless of scores above for concepts 1–4, this study should be scored as zero overall as the assets were not used for their purpose.**	1 = Community leads the mobilization of assets 0.66 = Community collaborates in the mobilization of assets 0.44 = Community is consulted about the mobilization of assets 0.22 = Community is informed about the mobilization of assets 0 = Community is not involved in the mobilization of assets or not applicable (no mobilization occurred)
*Asset prioritization [used to re-weight the other five elements]*	How much of the program or activities were focused on valuing or strengthening existing assets or identifying and supporting new assets for the agreed purpose? Denominator = estimate number of activities; numerator= estimate number of asset-based activities. If only some of one activity is asset based, divide by 2 (e.g. 20% becomes 10%). If it is not possible to estimate the proportion of activities, use the bracketed qualitative component or scale ends to indicate its relative prioritization to the overall program.	1 = All of it 0.9 0.8 = 80 per cent (most) 0.7 0.6 0.5 = 50 per cent 0.4 = (some) 0.3 0.2 = 20 per cent (little) 0.1 0 = None of it	

The first element is **reframing towards a fresh perspective** moving away from ‘what is wrong’ to the identification of opportunities and strengths (Foot and Hopkins, 2010). This involves taking a positive, appreciative approach that does not focus solely on problems or needs but on the potential of what is possible within the community. The reframed discussion within the community focuses on assets and understanding what is within the community, possibly untapped, that could be used. Reframing also involves identifying local champions who can lead and illustrate that change is possible.

The second element is **developing a common agenda.** Asset-based approaches recognize that there may be no set agenda initially, but this will develop over time. It is recognized that everyone within the community has a contribution to make. As trust is built there may be broader thinking about who could help the community to achieve its goals. A realistic and achievable agenda should develop when negotiation between communities and services about what is possible occurs.

Thirdly, **recognizing assets** that are present within the community. These assets include people, their skills and experiences. Ideally, this would be the first focus and use a ‘head, hands and heart’ approach to identify what the people in the community know, what they can do and what they care about (1); examples of assets have been reported elsewhere (Morgan and Ziglio, 2007; Foot and Hopkins, 2010). Assets can be analysed and expressed as: primary assets—those controlled by that community (e.g. community groups/associations); secondary assets—those controlled by those outside the community (e.g. GP practices, schools) and potential assets—assets outside a community that are controlled outside that community (e.g. grant-awarding bodies, colleges) (McKnight and Kretzmann, 1990). These assets may have been previously undiscovered or unused and this process makes them visible. The mapping may be undertaken at this stage, creating an inventory of assets (strengths, gifts and other resources), their connections and relationships, in preparation for using assets for local action and activity. Asset recognition done well will be undertaken and owned by community members and should take into account the community context (Kretzmann and McKnight, 1996).

The fourth element is **making connections to mobilize assets**; establishing the connections and growth and nurturing of assets and relationships. This important step enables the later mobilization. It builds on the skills and strengths of communities that become confident in their ability to be co-producers rather than recipients of services. This stage encourages the building of new relationships, strengthening and expanding existing relationships, as well as recognizing achievements within the community.

The final element is **mobilizing and co-producing assets and outcomes**; what needs to happen in order to bring about changes; i.e. what factors catalyse ‘having assets’ to mobilize them from the state in which they exist towards being ‘health-protective or health-promoting’. During mobilization, assets will be used for specific purposes; the community can see its assets and show how they connect and work to achieve positive outcomes.

### Developing the scoring system for the framework

A scoring system was developed for each of the elements within the framework. A scoring system of zero was chosen if the element was absent (and therefore the study for that element was aligned with a deficit approach); 0.22 if some of this element was reported, 0.66 if most of the element is present and 1.0 if the complete focus is covered in that element. When reviewing a paper, each element was scored using this system. After scoring each element, a decision was made about ‘asset prioritization’, i.e. how much of the study was focused on asset-based approaches (either valuing and/or strengthening existing assets, or identifying and supporting new assets for their agreed purpose). This prioritization was calculated by dividing the estimated number of asset-based activities in the study (nominator) by the overall number of activities (denominator). For example, a study that was completely (100%) asset-based would score 1, and a study with 40% asset-based activities would score 0.4 (some asset-based). This prioritization was used to re-weight the other five elements in the framework.

While an asset-based approach states that the community should be at the heart of the process (Foot and Hopkins, 2010), a positive score on any of the five core elements does not necessarily confirm that the community-led, or was even involved in, that particular process. This may be particularly pertinent for funded projects evaluating the effectiveness of public health interventions where a research team may drive implementation. After piloting, the assessment and scoring of the community involvement was a separate stage rather than being included within the evaluation of the five elements. It was impossible to position two separate aspects (whether an asset-based concept was present and how much community involvement was present) on a single continuum. A previously developed scale related to community engagement (community was leading, collaborating, consulting, informed, not involved) (Brunton *et al.*, 2014) was applied to each of the framework’s five elements. For the community engagement scoring, a scoring system from zero (community was not involved), 0.22 if the community is informed, 0.44 if the community is consulted, 0.66 if the community collaborates and 1.0 if the community leads the element.

Projects that do not mobilize assets, e.g. described asset identification but did nothing with them are scored as zero. This could be adapted if the process of asset discovery was the final point of interest by removing the elements in the framework that relate to asset mobilization.

### Framework testing

A total of 13 studies were tested. Five studies (Plescia *et al.*, 2008; Andrews *et al.*, 2012; Andersen *et al.*, 2013; Chen *et al.*, 2013; Woods *et al.*, 2013) were sampled from a review about community engagement for health (Brunton *et al.*, 2014), three studies (Baker *et al.*, 2007; Kegler *et al.*, 2009; Cinderby *et al.*, 2016) from a review of health assets in a global context (Van Bortel *et al.*, 2019) and five studies (McPhee *et al.*, 1995; Rogers *et al.*, 1995; Johnston *et al.*, 1998; Cheadle *et al.*, 2001; Stucki *et al.*, 2014) from our comparative review (Martin-Kerry *et al.*, 2019). Sampling deliberately aimed to include intervention studies where asset mobilization was likely, rather than studies that described asset identification only or a plan to develop an intervention. However, many of the studies only reported the asset identification, which is understandable given that once assets are identified, it may take the community time to mobilize them. Additionally, finding all the information can require the identification of all linked papers on a study to understand the full extent of an asset (or deficit) approach within a study. The extent to which each study described the five elements within our framework was measured and, for each element, how much community engagement was demonstrated.

Table 2 summarizes the individual scores for each element and the total score for each of the papers tested with the framework. The framework demonstrated that some studies were deficit-based (i.e. scored zero across all elements) (McPhee *et al.*, 1995; Andrews *et al.*, 2012; Andersen *et al.*, 2013; Chen *et al.*, 2013; Woods *et al.*, 2013; Stucki *et al.*, 2014). Some studies had some elements of an asset-based approach as seen by scores above zero in some elements (Rogers *et al.*, 1995; Johnston *et al.*, 1998; Cheadle *et al.*, 2001; Plescia *et al.*, 2008). Two of these studies did not report mobilization of the assets and so scored zero overall (Johnston *et al.*, 1998; Plescia *et al.*, 2008). Three studies showed strong elements of an asset-based approach (Baker *et al.*, 2007; Kegler *et al.*, 2009; Cinderby *et al.*, 2016).

**Table 2:** Testing the framework to see if we can distinguish between asset-based and deficit-based community studies

Study	Element 1	Community involvement	Element 2	Community involvement	Element 3	Community involvement	Element4	Community involvement	Element 5	Community involvement	Summary score (/10)	Prioritization	Total score
Andersen *et al.*, 2013	0	0	0	0	0	0	0	0	0	0	0	0	0
Andrews *et al.*, 2012	0	0	0	0	0	0	0	0	0	0	0	0	0
Baker *et al.*, 2007	1	0.66	0.66	0.66	1	0.66	0.66	0.66	0.66	0.66	7.28	1	7.28
Cheadle *et al.*, 2001	0	0	0.22	0.44	0	0	0.22	0.44	0.22	0.66	2.20	1.0	2.20
Chen Jr *et al.*, 2013	0	0	0	0	0	0	0	0	0	0	0	0	0
Cinderby *et al.*, 2016	0.66	0.44	0.66	0.66	0.66	0.66	1	0.66	0.66	0.66	6.72	1	6.72
Johnston *et al.*, 1998	0	0	0.22	0.44	0.22	0.22	0	0	0	0	0^*^	0.1 (approx.)	0^*^
Kegler *et al.*, 2009	0.66	0.44	0.22	0.66	0.22	0.66	0.66	0.66	0.66	0.66	5.50	1	5.50
McPhee *et al.*, 1995	0	0	0	0	0	0	0	0	0	0	0	0	0
Plescia *et al.*, 2008	0	0	0	0	0.22	0.22	0.66	0.66	0	0	0^*^	0.3	0^*^
Rogers *et al.*, 1995	0	0	0.22	0.44	0.22	0.22	0	0	0.22	0.22	1.54	1.0	1.54
Stucki *et al.*, 2014	0	0	0	0	0.	0	0	0	0	0	0	0	0
Woods *et al.*, 2013	0	0	0	0	0	0	0	0	0	0	0	0	0

^*^ No mobilization of assets therefore scores zero overall.

## DISCUSSION

This article describes the development of a framework that can be used to distinguish asset-based studies from studies using deficit-based approaches. Asset-based approaches are not prescriptive and value the community context within which it is based (McLean *et al.*, 2017). Identification of asset-based approaches has been difficult due to inconsistencies in the language used and studies may call themselves asset-based whilst being more deficit in orientation (Friedli, 2013). Synthesis of community-based projects for evidence of effectiveness is challenging and it can be unclear which components of a community-based project led to any effectiveness created by the intervention. This framework builds on work begun previously by other researchers looking at which elements of asset-based approaches lead to an asset-based intervention working (Cassetti *et al.*, 2020).

Synthesis of asset-based studies is further challenged by a diversity of organising models, their foci and the labelling of activities that might not accurately reflect the approach taken. It is anticipated that this framework will enable policymakers and researchers to distinguish studies that are asset-based from other community-based studies. Public health policy directions suggest that new ways of working with communities are needed. The value of communities supporting health and wellbeing, the importance of community connections, and lived experience are increasing (McLean *et al.*, 2017). Researchers in the asset-based approach field suggest that these approaches could assist in reducing inequities through strengthening social networks, empowering people to use assets within their community and increasing their capacity and control over their health (Foot and Hopkins, 2010). The studies that were identified as zero across all elements in the framework are defined as deficit-based studies. This categorization is reliable as there was no evidence of any asset-based approaches (reflected in the five elements of the framework) within these studies.

The flexibility of the framework may be of significance to researchers interested in studies taking an asset-based approach and who wish to distinguish between the approaches taken. The motivation for the framework was born from a need to synthesise effectiveness and focuses on interventional studies, but those who wish to identify elements of asset-based approaches in non-interventional studies could adapt the framework to assist this process. If researchers wanted to identify studies that took an asset-based approach but had not mobilized the assets, then earlier elements of the framework could be used to measure the degree of this. There are many studies that focus on asset identification but do not report the use of these within the study, which may happen much later. However, the study has still undertaken an asset approach.

There is a disproportionate amount of public health evidence available on how best to improve health that focuses on deficits as a starting point. There is increasing recognition about the importance of working in an asset-based way and valuing assets in public health practice (Morgan and Ziglio, 2007; Alvarez-Dardet *et al.*, 2015). The benefits of using strengths within the community to improve health have much potential and offer different opportunities to a deficit-based approach (McLean, 2011). It offers new solutions to promoting health and reducing health inequalities (Alvarez-Dardet *et al.*, 2015). However, much of the evidence around asset-based approaches is in case studies (McLean, 2011) and the evidence base for identifying assets and adopting an asset-based approach is limited (Morgan, 2014; Cassetti *et al.*, 2020). As the terminology of these approaches is likely to vary between location and studies, this framework offers an ability to identify studies using an asset-based approach that may not necessarily use associated terminology.

This framework is novel and offers a method to consistently review community-based studies to determine whether a study is more asset-based or deficit-based. It is aimed for use in public health interventions and has been tested on a range of community-based studies. Its effectiveness in differentiating between study types has been demonstrated through the scoring of 13 studies by one researcher. Through this process, a consistent approach to scoring has enabled the identification of studies that are more asset-based or more deficit-based in their approach, which suggests that policymakers and researchers will find the framework a useful tool in the identification of asset-based studies.

## CONCLUSION

This study is the first to the authors’ knowledge to develop a framework to distinguish between asset-based and deficit-based studies, acknowledging the variation in terminology and approaches within these studies. Given the increased focus on the value of asset-based approaches for public health interventions, this framework will be important for policymakers and researchers who wish to identify studies with more of an asset focus and to evaluate the impact of asset-based approaches. By distinguishing between studies that are more asset-based or deficit-based, researchers and policy-makers can use the framework to determine which elements of an asset-based intervention lead to change.
